# Mechanomedicine

**DOI:** 10.1007/s12551-018-0459-7

**Published:** 2018-09-29

**Authors:** Keiji Naruse

**Affiliations:** Department of Cardiovascular Physiology, Graduate School of Medicine, Dentistry and Pharmaceutical Sciences, Okayama University, 2-5-1 Shikata-cho, Kita-ku, Okayama, 700-8558 Japan

**Keywords:** Mechanobiology, Stretch-activated ion channel, SAKCA, TRPV2, Microfluidic sperm sorter, Hemostat

## Abstract

It has been a long time since the term mechanobiology became widely accepted, and broad research approaches, ranging from basic biology to medical research, have been conducted from the perspective of mechanobiology. Our group created the term “mechanomedicine” focusing on the field encompassing studies of the pathology and treatment of various diseases based on the knowledge obtained from mechanobiological studies and have promoted studies in this field. In the respiratory and cardiovascular systems, not only humoral factors but also physical factors such as contraction and expansion phenomena, and feedback from such phenomena to tissues and cells are important stimuli for maintaining homeostasis. Loss of homeostasis is considered to lead to pathological conditions. This review aims to provide an overview of mechanomedicine by introducing several mechanosensitive channels including one particular type of mechanosensor that we discovered in the cardiovascular system and by describing stretchable three-dimensional cell culture scaffolds using self-assembled peptides, a highly motile sperm sorter using a sperm sorting technique based on microfluidic mechanics, and a device to promote the development of fertilized ova.

## What is mechanomedicine?

Our group created the term “mechanomedicine” focusing on the field encompassing studies of the pathology and treatment of various diseases based on the knowledge obtained from mechanobiological studies and have promoted studies in this field (Naruse 2013, 2017a, b, 2018). Previous studies have widely investigated responses to mechanical stimuli exerted on tissues and cells, such as stretch, shear stress, and hydrostatic pressure. These include the mechanisms that convert mechanical stimuli to electrical signals in the sensory systems. Examples such as touch and hearing and the response of a bronchus or the myocardium to mechanical stimuli have been evaluated at the intracellular and tissue levels. Other commonly studied topics at the cellular level include phenomena associated with cell movement and chemotaxis in the cytoskeleton and focal adhesions. At the single molecule level, structural changes caused by applying stress to protein and changes in the molecular function of stretch-activated channels, enzyme activity, have also been reported. As described above, mechanobiological studies have been steadily progressing. The concept of mechanomedicine is derived from mechanobiology and was developed to encompass pathological understanding and treatment of diseases. Our group has conducted studies on the pathology and treatment of not only cardiovascular and respiratory diseases but also motor system and reproductive diseases. These studies are important for both conventional medicine and regenerative medicine.

## Mechanosensors

Although many molecular aspects of mechanosensors most upstream of mechanotransduction remain unknown, only mechanosensitive channels are well-understood. Numerous studies have directly measured single ionic currents activated by stretching the plasma membrane using the patch-clamp technique. The subjects in typical studies, including those involving our group, include a nonselective cation channel Mid1 (yeast) (Kanzaki et al. 1999), intracellular calcium-dependent stretch-activated potassium channel (SAKCA; myocardium, described later) (Kawakubo et al. 1999; Naruse et al. 2009), and transient receptor potential vanilloid isoform 2 (TRPV2; endothelial cell and myocardium, described later) (Katanosaka et al. 2007; Katanosaka et al. 2014). Other groups have reported a large-conductance mechanosensitive ion channel (M_sc_L, *Escherichia coli*) (Martinac et al. 1987, 1990), Piezo type (Coste et al. 2012), etc. Additionally, it has been suggested that focal adhesion proteins are mechanosensors (Wang et al. 1993).

### Cardiovascular system

#### Endothelial cells

**Fig. 1:**
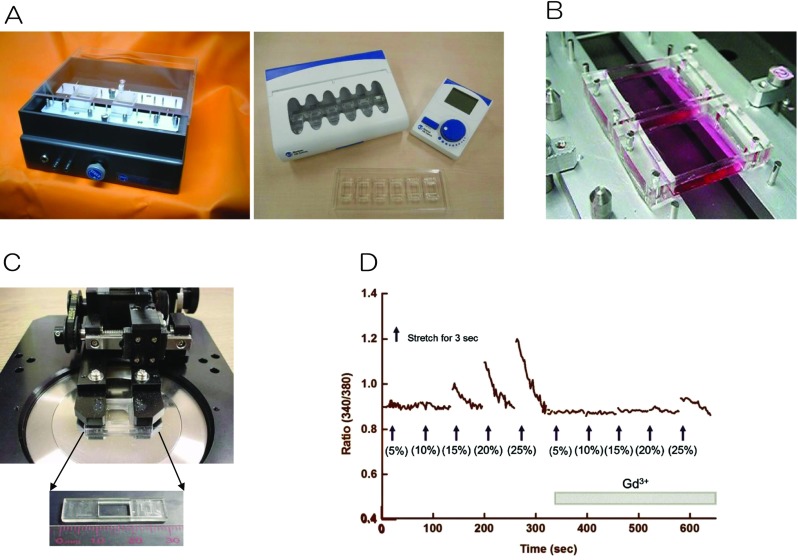
**a** Stretch apparatus: left, manufactured by Strex, Inc. and right, Menicon Life Science. **b** The stretch chambers composed of extendable transparent silicone resin (polydimethylsiloxane, PDMS). **c** Stretch apparatus for microscope experiment. **d** [Ca^2+^]_i_ changes in response to stretch

Blood vessels are constantly exposed to mechanical stimuli, such as shear stress from blood flow, hydrostatic blood pressure, and pulsatile stretch due to systolic-diastolic fluctuations. In response to these stimuli, endothelial and smooth muscle cells secrete vasoactive substances to maintain tonus, express genes and proteins, and cause morphological changes. Disruption of these mechanisms may cause various diseases, such as hypertension and arteriosclerosis. We developed and marketed devices that apply a stretching stimulus to cultured cells (Fig. 1a). The stretch chamber consists of a cell culture unit composed of extendable transparent silicone resin (polydimethylsiloxane, PDMS). It has a membrane approximately 100 μm thick, coated with an extracellular matrix protein, such as fibronectin. Endothelial cells are cultured in the chamber (Fig. 1b). Using a microscope-compatible stretching device (Fig. 1c), a 20% stretch stimulus is applied for 3 s. As a result, the intensity-dependent intracellular Ca^2+^ level transiently increases and the mechanosensitive channel is blocked with Gd^3+^ (Fig. 1d).

It is suggested that TRPV2 channels in endothelial cells function as a mechanosensor to transport Ca^2+^ in a stretch-dependent manner. Stretching human embryo kidney (HEK) cells only minimally increased stretch-dependent Ca^2+^ levels, whereas stretching TRPV2-expressing HEK cells increased Ca^2+^ levels to a greater extent. Moreover, in human umbilical vein endothelial cells (HUVECs), increases in stretch-dependent Ca^2+^ levels disappear by including a small interfering ribonucleic acid (siRNA) targeting TRPV2 channel (Katanosaka et al. 2007).

Various studies have also evaluated intracellular signal transduction in endothelial cells stimulated by stretching (mechanotransduction). Endothelial cells are aligned perpendicular to the direction of the stretch (Naruse and Sokabe 1993). During this process, protein tyrosine phosphorylation of focal adhesion kinase (FAK) is introduced, which is dependent on increases in intracellular Ca^2+^ levels. When phosphorylation of FAK was inhibited or blocked, stretch-dependent alignment was also inhibited. Thus, FAK itself was shown to be important for this alignment (Yamada et al. 2000). Furthermore, stretching stimulation increases intracellular levels of cyclic adenosine monophosphate (cAMP) (Yamada et al. 2000) and causes the production of superoxide (Aikawa et al. 2001) and nitric oxide (NO) (Takeda et al. 2006), as well as activating nuclear factor kappa B (NFκB) (Kobayashi et al. 2003).

#### Myocardium

**Fig. 2:**
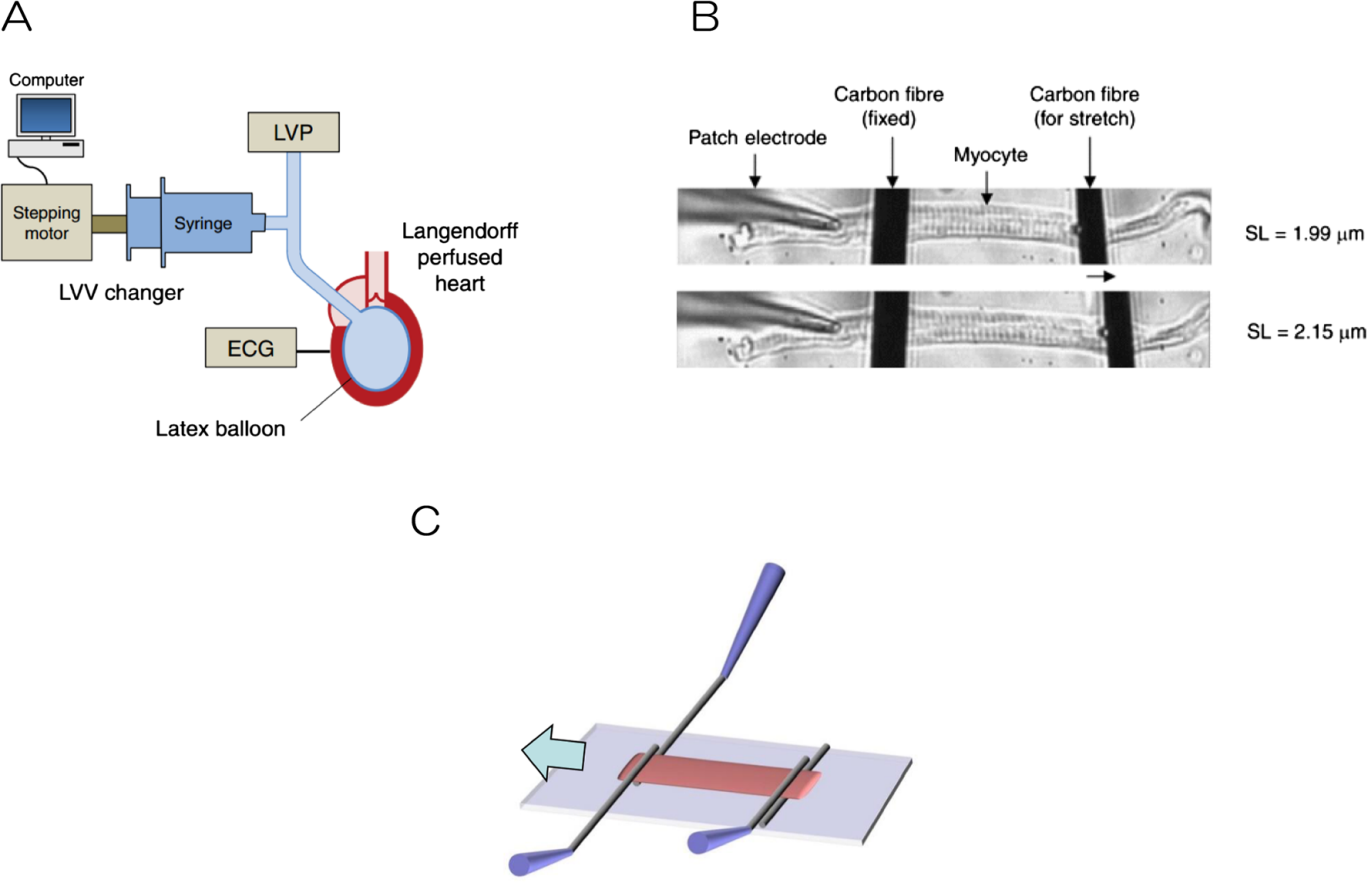
**a** Intraventricular balloon expansion using Langendorff-perfused samples. **b** Method using carbon fiber electrodes and isolated cardiomyocytes to measure myocardial tension and electrophysiological experiments. **c** A new stretching method that allows expansion of the myocardium up to physiological sarcomere length

The heart is a dynamic organ that repeatedly contracts and relaxes. This activity is induced by electrical excitation, a mechanical-electrical-feedback mechanism also controls the electrical features of contraction and expansion (Kohl and Sachs 2001). The myocardia also contain various mechanosensitive channels (Bustamante et al. 1991). Depending on contraction and expansion, the myocardia are activated and the resulting ionic changes alter the states of contraction and expansion. Our group established a model of stretch-dependent arrhythmia induced by Iribe et al. (2011) using a carbon fiber electrodes and isolated cardiomyocytes (Fig. 2a) to measure tension and electrophysiological activity (Fig. 2b) (Iribe et al. 2013). Additionally, we recently developed a new stretching method that allows expansion of the myocardia up to the physiological sarcomere length experienced in the heart (Fig. 2c) (Iribe et al. 2014).

#### Mechanosensitive channels

**Fig. 3:**
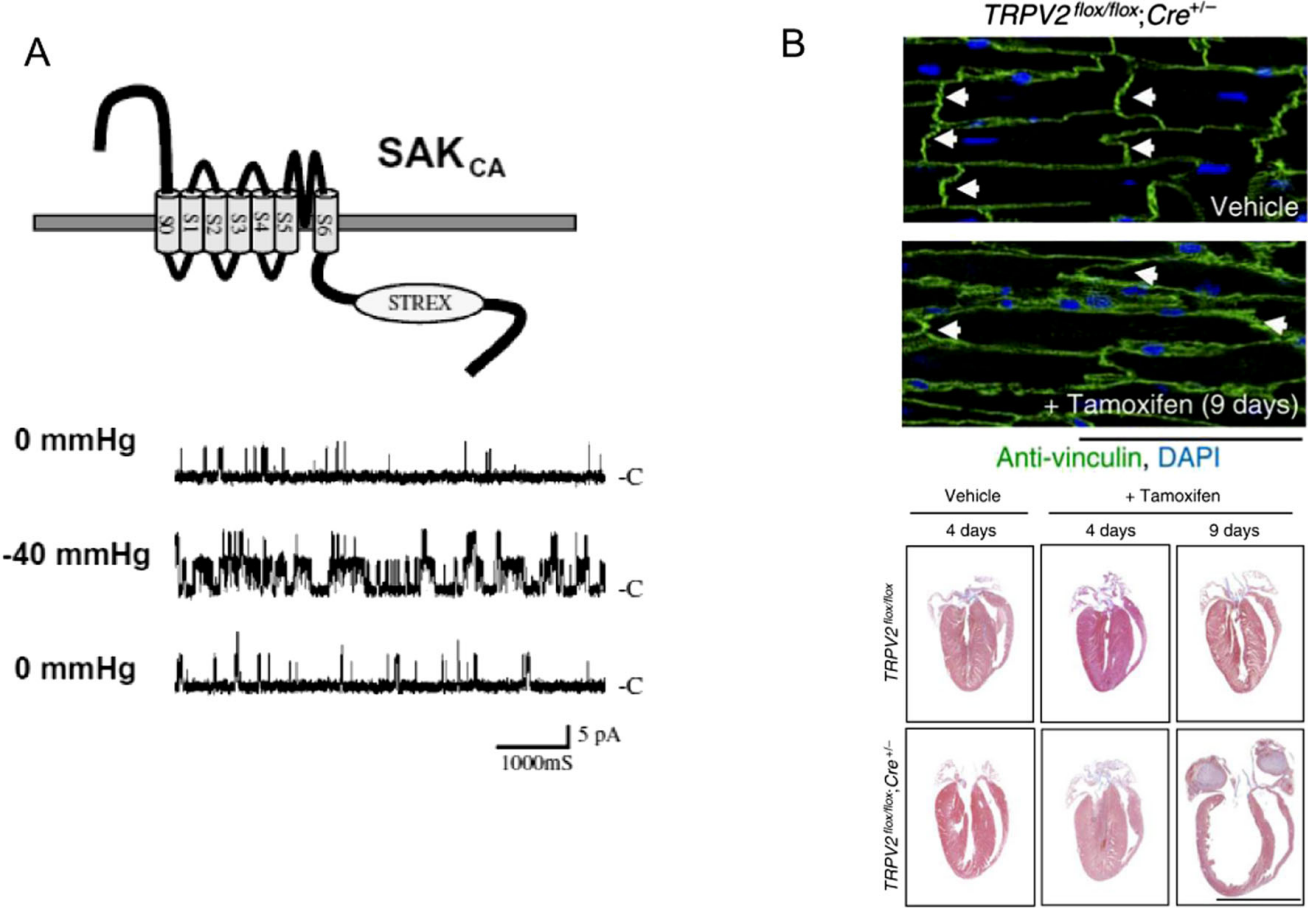
**a** STREX sequence was found to be important for stretch activation in SAKCA. **b** Nine days after administration of Tamoxifen, the intercalated disks collapsed (upper), causing ventricular dilatation (lower)

SAKCA (stretch-activated Ca^2+^-activated K^+^ channel) is a K^+^-permeable mechanosensitive channel present in the cultured chick ventricular cardiac muscle. Interestingly, it is activated by intracellular Ca^2+^ and ATP (Kawakubo et al. 1999). Thus, we cloned the channel using degenerate primers against BKCa channel from the chick embryonic heart cDNA library and found out that the channel contains the STREX (stress-axis regulated exon) sequence. This sequence is important for stretch activation (Fig. 3a) (Naruse et al. 2009). In the heart, TRPV2 channels exist in the intercalated disks. During systole, these disks are stretched in the direction of the force. We generated TRPV2 conditional knockout mice to analyze their phenotypes (Fig. 3b) (Katanosaka et al. 2014). Nine days after administration of tamoxifen to induce Cre recombinase, nearly all TRPV2 disappeared from the intercalated disks which simultaneously collapsed, causing ventricular dilatation. Cardiac pump function was also reduced and nearly all mice died 1 week later. When isolated newborn myocardial cells were cultured, stretch stimulation did not increase intracellular Ca^2+^ levels. Additionally, cell morphology was abnormal. Interestingly, these mice were rescued by administering insulin-like growth factor 1 (IGF-1). This suggests that increases in intracellular Ca^2+^ levels following activation of TRPV2 is associated with the phosphatidylinositol 3-kinase (PI3K)/protein kinase B (AKT) pathway.

### Other studies in mechanomedicine

**Fig. 4:**
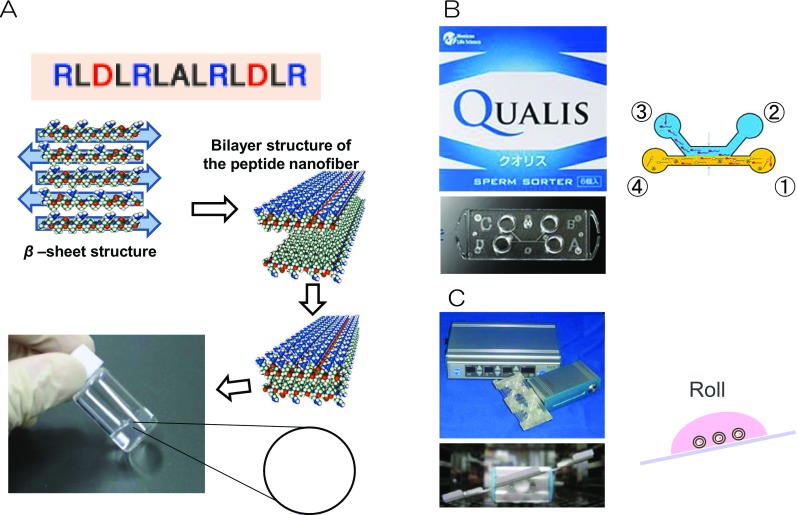
**a** A molecular model of self-assembling peptide SPG-178 and a diagram of the formation of the hydrogel from the peptide monomer. **b** The Microfluidic motile sperm sorter Qualis® is composed of four numbered chambers ①, ②, ③, ④, and a micro channel connected to each chamber. After applying sperm sorting medium to chambers ②, ③, ④ and a semen sample to chamber ① in an appropriate amount, two streams of fluid with laminar flow can be formed in parallel (① → ④, ② → ③). Only motile spermatozoa are allowed to swim into the parallel stream, followed by being isolated from chamber ③. **c** Tilting embryo culture system. This device consists of a control unit and a motor unit with a tilting plate with four-well chambers. Mouse embryos were cultured at a tilt angle of 20° with a holding time of 1 min. It was then rotated at a tilt angle of − 20° with a holding time of 1 min, and the cycle was repeated

To apply stretching and relaxing stimuli to motor organs (e.g., skeletal muscles), as well as cartilage, and bone, stretchable three-dimensional cell culture scaffold was developed that was fully synthesized, self-assembled peptide that substituted for animal-derived collagen (Nagai et al. 2012). Importantly, this peptide acts as a hemostatic drug (Fig. 4a) (Komatsu et al. 2014). Also, we developed a highly motile sperm sorter using a technique based on microfluidic mechanics (Fig. 4b) (Matsuura et al. 2012. This device promotes the development of fertilized ova by applying shear stress generated in the Fallopian tubes where the ova are fertilized (tilting embryo culture system, TECS) (Fig. 4c) (Matsuura et al. 2010). These devices are currently being used in clinical practice (Hara et al. 2013).

The above mechanomedicine techniques have been used in studies of the cardiovascular and other systems, and they have achieved favorable results. The information presented in this review could prove valuable to researchers in these fields.

## Conclusion

In Japan, mechanobiological studies have been promoted through grants, such as the Grant-in-Aid for Specially Promoted Research in the Grants-in-Aid for Scientific Research and the International Cooperative Research Project (ICORP) of the Japan Science and Technology Agency. During 2010–2012, mechanobiology was included in the list of disciplines and research fields with a time limit. Thus, mechanobiology became a new science and field of technology in Japan. Mechanobiology integrates physics, engineering, medicine, and biology, making active collaboration with researchers from different fields essential.

We hope that the active involvement of many researchers and clinicians in this mechano field will lead to the reconsideration of studies, diagnoses, and treatment of various diseases.
